# Optimized detection of ESBL producers, carbapenemase producers, and VRE from rectal swabs using enrichment broth for superior screening sensitivity

**DOI:** 10.1007/s10096-025-05288-1

**Published:** 2025-09-26

**Authors:** Dominique S Blanc, Massiha Kamyar, Patrice Nordmann, Laurent Poirel, Gilbert Greub

**Affiliations:** 1 Infection Prevention and Control Unit, Infectious Diseases Service, Lausanne University Hospital and University of Lausanne, Lausanne, Switzerland; 2 Swiss National Reference Center for Emerging Antibiotic Resistance, Fribourg, Switzerland; 3 Institute of Microbiology, Lausanne University Hospital and University of Lausanne, Lausanne, 1005 Switzerland; 4 Medical and Molecular Microbiology, University of Fribourg, Fribourg, Switzerland

## Abstract

This study evaluates the added value of using enrichment broths for improving the detection of multidrug-resistant (MDR) bacteria—VRE, ESBL producers, and carbapenemase-producing organisms (CPOs)—from rectal swabs. Comparing direct plating to enrichment-based methods significantly increased detection sensitivity, especially for CPOs (from 17 to 27 cases, a 58.8% increase). Detection rates for ESBL producers and VRE also improved significantly (by 20%). Pre-enrichment is therefore a valuable addition to routine MDR bacteria screening protocols, which is particularly useful when high accuracy is needed for prevention and infection control as well as for outbreak management.

## Text

Multidrug-resistant (MDR) bacteria represent a growing global health concern, significantly increasing morbidity and mortality due to the limited availability of effective treatment options [1]. Early detection of patients colonized with MDR bacteria is critical for implementing timely infection control measures and preventing nosocomial outbreaks [2–6]. However, optimal screening strategies for MDR bacterial colonization remain a subject of ongoing investigation.

Many healthcare institutions incorporate MDR bacterial screening into their infection control protocols, particularly for extended-spectrum β-lactamase (ESBL) producers, carbapenemase-producing organisms (CPO), and vancomycin-resistant enterococci (VRE). However, there is limited evidence on the most effective methodologies to maximize sensitivity in routine clinical settings [7].

Conventional screening methods rely on direct plating onto chromogenic and antibiotic-containing media, which have demonstrated improved sensitivity and specificity in detecting MDR bacteria from stool and rectal swab samples [1, 2, 8–11]. Enrichment broths have the potential to improve the detection of low-abundance bacterial populations by promoting their selective growth before plating. Although evidence supports their utility in outbreak and research settings [5, 7, 8, 12], their value in real-life, routine MDR surveillance workflows remains under-evaluated, especially in terms of detection gains across all three major MDR groups.

This study aims to evaluate the added value of incorporating optimized enrichment broth protocols into routine hospital screening for VRE, ESBL-producing bacteria, and carbapenemase-producing organisms (CPO) using prospectively collected rectal swabs. It builds directly on our previous in vitro study [13], which identified the most effective enrichment broth compositions for each target group using spiked stool samples. While several studies have demonstrated the potential of enrichment approaches, clinical validation under real-world conditions remains limited. Here, we address this gap by applying the optimized protocols to a high-volume, routine hospital screening program and systematically comparing enrichment-based detection to conventional direct plating.

The University Hospital of Lausanne is a tertiary care hospital with 1,100 beds. The MDR screening infection control policy follows Swiss recommendations (www.swissnoso.ch), including weekly screening of high-risk patients and screening of patients transferred from abroad. Screening for VRE, ESBL, and CPO was performed on rectal swabs containing 1 mL of Amies transport medium (eSwab, Copan, Italy). The swab was manually streaked onto different culture media (direct plating) (VRE brilliance, Oxoid, ThermoFisher; BSLE CHROMagar, France; and SuperCARBA, CHROMagar, France). Then, 0.25 mL of the sample was inoculated into each enrichment broth. Enrichment broths consisted of trypticase soy broth (TSB) with antibiotics (Table 1). Plates were read after 24 h of incubation. After overnight incubation, enrichment broths were subcultured onto the same media used for direct plating. Culture plates were incubated for 24 h before being read. Incubation was carried out under normal atmospheric conditions at 37 °C. The study was conducted according to the laboratory’s routine schedule (Monday to Friday); samples received on Fridays were partially processed before the weekend, with final readings and subcultures completed on Monday, while samples received during the weekend were processed on the next working day.

**Table 1:** Composition and Preparation of enrichment broths

MDR	Stock Antibiotic Solution*	Broth Preparation	Final Concentration
VRE	--	Place a 5 µg vancomycin disk in a tube containing 5 mL of TSB	1 µg/mL
ESBL	Place 10 disks of 5 µg cefotaxime in 10 mL of 0.9% NaCl in a glass tube**	Add 100 µL of the cefotaxime stock solution to each 5 mL TSB tube	0.1 µg/mL
CARBA	Place 5 disks of 10 µg ertapenem in 10 mL of 0.9% NaCl in a glass tube**	Add 100 µL of the ertapenem stock solution to each 5 mL TSB tube	0.1 µg/mL

* Stocks of antibiotic solutions were prepared every day

**Shake vigorously, let stand for 30 min, then shake vigorously again

All suspected colonies from either direct plating or subcultures were identified using MALDI-TOF. Resistance to vancomycin in enterococci was confirmed by detecting the *vanA/vanB* genes (GeneXpert, Cepheid). ESBL resistance in enterobacteria was confirmed using the NG CTX-M test Multi (NG Biotech, France) and by detecting synergy between ceftriaxone and amoxicillin-clavulanic acid. The production of carbapenemases in enterobacteria and *Pseudomonas* sp. was confirmed using the NG CARBA test (NG Biotech, France) and in *Acinetobacter s*p. using the Resist Acineto test (Coris BioConcept, Belgium).

Between August 2023 and March 2024, most rectal swabs submitted to our laboratory for MDR screening were included in the study. Table 2 presents the number of samples processed and the corresponding number of positive results. Overall, the inclusion of enrichment broths in the screening analysis significantly improved sensitivity of detection, increasing it from 20% for VRE and ESBL to 58.8% for CPO.

**Table 2:** Results of screening samples for VRE, ESBL and CPO

MDR	No samples	Negative	Positive (all)	Positive (direct plating)	Positive (additional cases detected by enrichment)	Increase in sensibility (%)
VRE	1402	1378	24	20	4	20.0
ESBL	1049	994	55	45	10*	22.2
CPO	1092	1065	27	17	10	58.8

### VRE screening

*Enterococcus faecium* carrying the *vanA* gene was the predominant organism identified. Only one isolate of *E. faecalis vanA* positive was detected, and no *vanB*-positive enterococci was found. All four samples that tested positive only after enrichment broth were *vanA*- positive *E. faecium*. These findings are consistent with previous studies demonstrating increased sensitivity when an enrichment broth was used [14–16]. To accelerate time to results, several studies have employed PCR directly on the enrichment broth to detect *vanA* and *vanB* genes. Compared to direct culture, these approaches consistently showed significantly improved sensitivity for VRE detection [2, 17].

### ESBL screening

A total of 45 *Klebsiella pneumoniae*, 3 *Klebsiella oxytoca*, 3 *Klebsiella aerogenes*, 2 *Enterobacter cloacae*, 3 *Citrobacter koseri*, and one isolate each of *Serratia fonticola*, *Providencia stuartii*,* Morganella morganii*, and *Citrobacter freundii* were identified. In five samples, *K. pneumoniae* was found in association with another ESBL-producing species. Among the ten samples that tested positive only using an enrichment broth, eight contained *K. pneumoniae*, one *K. aerogenes*, and one *S. fonticola*. All nine *Klebsiella* isolates were CTX-M positive. For *S. fonticola*, ESBL production was confirmed by synergy testing between ceftriaxone and amoxicillin-clavulanic acid. These findings align with previous reports. For example, Jazmati et al. [18]. demonstrated that 31.7% of ESBL carriers were identified only after a pre-enrichment step. Similarly, Blane et al. [19]. showed that pre-enrichment increased the sensitivity of ESBL detection by 25–30%, but extending the incubation period to 48 h did not improve detection rates and instead led to increased growth of non-ESBL organisms.

### CPO screening

Among the CPO isolates, *Escherichia coli* was the most frequently identified species (*n* = 12), followed by *Klebsiella pneumoniae* (*n* = 11), *Acinetobacter baumannii* (*n* = 3), *Proteus mirabilis* and *Pseudomonas aeruginosa* (*n* = 2 each), and *Citrobacter freundii* (*n* = 1). In three samples, two to three different CPO species were detected simultaneously. The most commonly identified carbapenemase was OXA-type (*n* = 17), comprising OXA-48 (*n* = 9), OXA-181 (*n* = 4), OXA-244 (*n* = 2), and OXA-23 (*n* = 2). This was followed by NDM (*n* = 7), KPC (*n* = 5), VIM (*n* = 3), and IMP (*n* = 1). One isolate harbored two different carbapenemase genes. Among the ten CPO-positive samples detected only after enrichment broth, eight contained *E. coli* producing NDM or OXA enzymes (three OXA-48, two OXA-244, one OXA-181, and two NDM). The remaining two were *A. baumannii* (NDM) and *K. pneumoniae* (KPC). In two cases, enrichment also facilitated the recovery of an additional CPO species. These results align with previous studies. For example, Glaser et al. [20]. reported that direct inoculation of chromID CARBA plates detected only 53% of CRE cases, whereas the addition of a meropenem enrichment broth increased the detection rate to 88% and uncovered additional CRE-positive samples. Similarly, Ciesielczuk et al. [21]. showed that enrichment with a temocillin-containing broth increased the recovery of OXA-48-producing CPE from 3% to 12% during an outbreak investigation focused on OXA-48-producing strains.

This study evaluates the implementation of optimized enrichment broth protocols in a real-life, high-volume clinical screening context. Whereas our previous study demonstrated a proof-of-concept in vitro, the current work validates these findings using a large, prospectively collected sample set, demonstrating the method’s feasibility and enhanced performance under routine laboratory conditions. It demonstrates that the use of pre-enrichment broths significantly enhances the detection of VRE, ESBL, and CPO in surveillance samples.

A key limitation is that the study was conducted at a single center, making it subject to the influence of local MDR epidemiology. However, as the formulation of the enrichment broths was previously optimized in vitro using a wide range of MDR strains [13], we expect these findings to be reproducible worldwide. Another potential limitation of this study is that the quality of the rectal swab samples was not systematically assessed prior to processing. All swabs were accepted regardless of whether visible fecal material was present. While this approach reflects the operational reality of many surveillance programs, particularly given the difficulties of obtaining rectal swabs in some patient populations, it may have contributed to a lower sensitivity for detecting low-level colonization in primo culture. The impact of sample quality on detection rates remains an open and important question in clinical practice, and further studies are warranted to evaluate whether stricter quality control measures (e.g., rejection of unsoiled swabs) would significantly enhance diagnostic performance.

In conclusion, incorporating a pre-enrichment step into the MDR screening workflow markedly increases sensitivity. This approach is particularly valuable in settings where high diagnostic accuracy is essential, such as infection control surveillance and outbreak investigations. However, it increases the time to result by an additional day, which might also be crucial in such situations.

## Data Availability

No datasets were generated or analysed during the current study.
